# An Efficient Deep Learning Method for Detection of COVID-19 Infection Using Chest X-ray Images

**DOI:** 10.3390/diagnostics13010131

**Published:** 2022-12-30

**Authors:** Soumya Ranjan Nayak, Deepak Ranjan Nayak, Utkarsh Sinha, Vaibhav Arora, Ram Bilas Pachori

**Affiliations:** 1 Amity School of Engineering and Technology, Amity University Uttar Pradesh, Noida 201301, India; 2 Department of Computer Science and Engineering, Malaviya National Institute of Technology, Jaipur 302017, India; 3 Department of Electrical Engineering, Indian Institute of Technology Indore, Indore 453552, India

**Keywords:** COVID-19, LW-CORONet, CNN, transfer learning, chest X-ray

## Abstract

The research community has recently shown significant interest in designing automated systems to detect coronavirus disease 2019 (COVID-19) using deep learning approaches and chest radiography images. However, state-of-the-art deep learning techniques, especially convolutional neural networks (CNNs), demand more learnable parameters and memory. Therefore, they may not be suitable for real-time diagnosis. Thus, the design of a lightweight CNN model for fast and accurate COVID-19 detection is an urgent need. In this paper, a lightweight CNN model called LW-CORONet is proposed that comprises a sequence of convolution, rectified linear unit (ReLU), and pooling layers followed by two fully connected layers. The proposed model facilitates extracting meaningful features from the chest X-ray (CXR) images with only five learnable layers. The proposed model is evaluated using two larger CXR datasets (Dataset-1: 2250 images and Dataset-2: 15,999 images) and the classification accuracy obtained are 98.67% and 99.00% on Dataset-1 and 95.67% and 96.25% on Dataset-2 for multi-class and binary classification cases, respectively. The results are compared with four contemporary pre-trained CNN models as well as state-of-the-art models. The effect of several hyperparameters: different optimization techniques, batch size, and learning rate have also been investigated. The proposed model demands fewer parameters and requires less memory space. Hence, it is effective for COVID-19 detection and can be utilized as a supplementary tool to assist radiologists in their diagnosis.

## 1. Introduction

The COVID-19 pandemic has caused severe healthcare crises across the globe in a very short period. The pandemic broke out in early December 2019 in Wuhan, China, and was declared a global pandemic on 11th March 2020 by the World Health Organization (WHO) [1]. Researchers across the world have reported COVID-19 to be a highly infectious disease that severely affects the respiratory system and has common symptoms like dry cough, myalgia, fever, headache, chest pain, and sore throat [2]. The current medical diagnostic processes lack proper medicine and drugs as well as hospital resources for the treatment of COVID-19 infection [3,4]. Reverse transcription-polymerase chain reaction (RT-PCR), a manual, time-consuming, and costly tool, is the most frequently used diagnostic method for detection which causes the risk to medical staff [5,6]. It is still an ongoing pandemic and has led to various variants, thereby, resulting in high mortality rates in many countries. Hence, there is a strong need for a safe and effective methodology that can detect COVID-19 infection at an early stage. The significance of two imaging modalities such as chest X-ray (CXR) and computed tomography (CT) has been studied for diagnosing COVID-19 [7,8,9]. However, manual visual inspection of both CXR and CT images is time-taking and tedious which may sometimes result in an inaccurate diagnosis [7,10]. Recently, tremendous efforts have been made in developing artificial intelligence (AI) based automated models for accurate diagnosis of COVID-19 to lessen the workload of radiologists [11].

Deep learning (DL) algorithms, especially, convolutional neural network (CNN), have offered efficient solutions for pneumonia detection in CXR images [12,13,14,15,16]. Soon after, many DL-based approaches have been reported to diagnose COVID-19 using CXR images. Ozturk et al. [17] proposed the DarkCovidNet model for COVID-19 detection in CXR images which obtained a classification accuracy of 98.08% and 87.02% for two-class and three-class scenarios, respectively. Hemdan et al. [18] proposed COVIDX-Net for binary classification task which was validated using only 50 CXR images. Narin et al. [19] proposed a model using the ResNet-50 and achieved a binary classification accuracy of 98% over 100 images. Ucar and Korkmaz [20] presented a multi-class classification system based on SqueezeNet and Bayesian optimizer that yielded an accuracy of 98.3%. Rahimzadeh and Attar [21] designed a model that concatenates Xception and ResNet50 networks and yields 91.4% accuracy for multi-class cases. Wang et al. [22] developed a tailored deep CNN model using CXR images that achieved an accuracy of 93.3%. Toğaçar et al. [23] achieved a classification accuracy of 97.06% using fuzzy color, stacking approach, and two DL approaches such as MobileNetV2 and SqueezeNet. Toraman et al. [24] proposed a convolutional CapsNet model to detect COVID-19 using CXR images and achieved an accuracy of 84.22% for multi-class classification cases. Han et al. [25] developed a deep 3D multiple instance learning methodology using chest CT images and obtained an accuracy of 94.3% on a three-class classification task. Zhang et al. [26] proposed a 7-layer-based CNN with stochastic pooling to detect COVID-19 from CT images. Wang et al. [27] developed a novel CCSHNet system for COVID-19 classification where two best pre-trained CNN models were selected to learn features and a discriminant correlation analysis method was used to fuse those features. Chaudhary and Pachori [28] used pretrained CNN models over the sub-band images generated from ourier-Bessel series expansion-based decomposition (FBSE) for COVID-19 detection. Joshi et al. [29] designed a multi-scale CNN for effective COVID-19 diagnosis from CT images. Recently, Bhattacharyya et al. [30] employed the VGG-19 model with the binary robust invariant scalable key points (BRISK) to detect COVID-19 cases from X-ray images. Jyoti et al. [31] proposed an automated COVID-19 detection method using memristive crossbar array-based tunable Q-wavelet transform (TQWT) and ResNet-50.

The literature shows that many existing DL-based methods have been validated using a few annotated CXR/CT images for COVID-19 diagnosis. The most frequently used DL models require a lot of training parameters and memory. The design of a lightweight DL method thus assumes significant urgency. Moreover, the performance of DL models relies on several hyperparameters: learning rate, batch size, type of optimizer, and the number of epochs, etc. However, there are only a limited number of studies on these parameters. Hence, there exists ample scope to conduct an investigation that includes an in-depth analysis of different hyperparameters to obtain the best possible results for COVID-19 detection. In a recent study [32], the COVID-19 classification performance was evaluated using eight pre-trained CNN models over a small dataset. All those models required a huge number of parameters. The performance of these models was evaluated for only binary classification scenarios. Nonetheless, despite being very challenging, a multi-class classification is in high demand.

To address the aforementioned issues, a lightweight CNN is proposed for the automated detection of COVID-19 infection in the current study. The major contributions of this study are summarized as follows:

- A light-weight CNN model is proposed to diagnose COVID-19 infection in CXR images which require low computational cost and memory, thereby making it more suitable for real-time diagnosis.
- The impact of several hyperparameters like different optimization techniques, number of epochs, batch size, and the learning rate is analyzed.
- The performances of both binary (Normal and COVID-19) and multi-class (Normal, COVID-19, and pneumonia) classification cases have been studied.
- A performance evaluation is conducted employing two larger datasets. Finally, the proposed model is compared with a few contemporary pre-trained CNN architectures including VGG-19 [33], ResNet-101 [34], DenseNet-121 [35], and Xception [36] in terms of the number of parameters and memory required. The performance of the model is also compared with a few state-of-the-art methods.

The remaining of this article is structured as follows: In Section 2, the datasets used and the suggested method for automated COVID-19 diagnosis are detailed. Section 3 presents the experimental setup and results. Finally, Section 4 draws the concluding remarks.

## 2. Materials and Methodology

This section exhaustively elucidates the dataset used in the current investigation and the proposed approach for the classification of various lung diseases. In addition, it delineates a few contemporary CNN architectures which are used for comparison purposes.

### 2.1. Datasets Used

The first dataset (Dataset-1) considered in our study comprises CXR images of three classes: Normal, COVID-19, and pneumonia that were collected from Figshare repository https://figshare.com/articles/COVID-19_Chest_X-RayImage_Repository/12580328, accessed on 21 November 2022 [37]. The quantity of available radiography images of COVID-19 patients is still limited. In addition, data imbalance has remained a major concern. Therefore, a total of 2250 CXR samples were collected for the three classes with 750 samples in each class. Dataset-1 mainly considered the COVID-19 cases from several open sources which are updated periodically.

**Figure 1:**
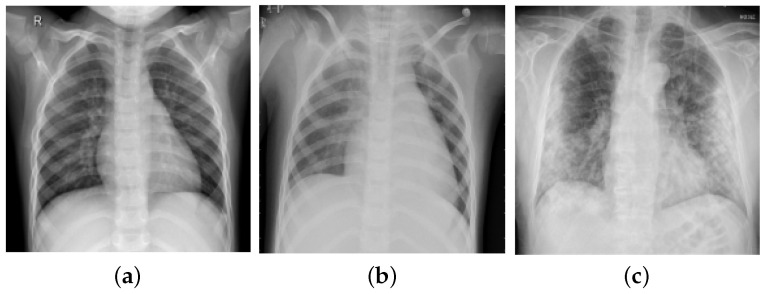
Frontal-view CXR samples of three categories: (**a**) Normal, (**b**) Pneumonia, and (**c**) COVID-19.

To further verify the efficacy of the proposed model, we considered another dataset COVIDx-V7A (termed as Dataset-2 in this study) which comprises 15,999 CXR images from 15,122 patients across 51 countries and is the largest open-access dataset available to date [22]. These images were collected from five different data repositories and are available at https://github.com/lindawangg/COVID-Net, accessed on 21 November 2022. Table 1 shows the detailed specification of the considered datasets. The samples of a few frontal-view CXR images of different classes from Dataset-1 are presented in Figure 1.

### 2.2. Proposed Methodology

**Figure 2:**
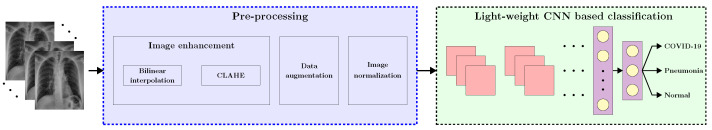
Block diagram of the proposed approach for automated detection of COVID-19 infection.

The proposed automated framework to classify COVID-19 infection cases from normal and pneumonia using CXR images are depicted in Figure 2. The framework consists of two stages: (i) prepossessing which includes image enhancement, data augmentation, and image normalization, and (ii) classification using the proposed light-weight CNN model (LW-CORONet). The detailed elucidation of each stage is given below.

#### 2.2.1. Preprocessing

It is one of the most critical stages in the proposed approach. This section describes each technique deployed in this stage.

**Figure 3:**
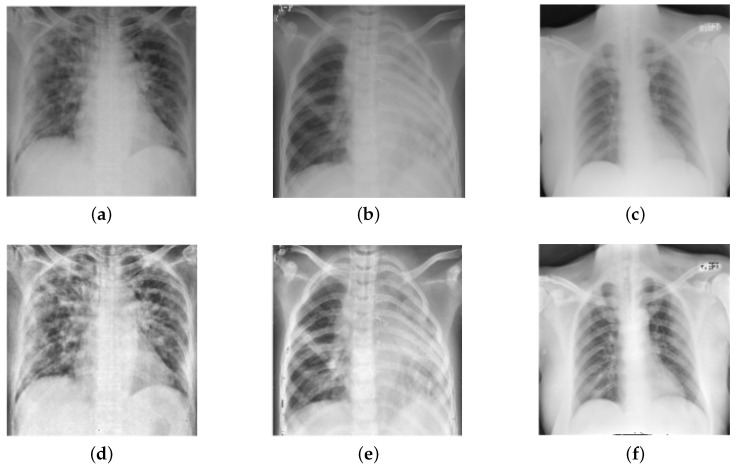
Results of image enhancement (BI and CLAHE): (**a**–**c**) indicate original images, (**d**–**f**) indicate enhanced images.

**Image Enhancement:** Image enhancement is frequently used in the biomedical image processing domain to improve image quality [38,39]. Therefore, in this study, we performed the image enhancement by employing bilinear interpolation (BI) followed by the contrast-limited adaptive histogram equalization (CLAHE) technique. Initially, BI was applied to replace the missing pixel by computing a weighted average of the nearest boundary pixels [40]. Then, CLAHE was employed for contrast enhancement that prevents over-enhancement of noise present in the images [41,42], thereby, facilitating better diagnosis. Figure 3 depicts the outcomes of the preprocessing stage (BI and CLAHE).

**Data Augmentation:** The CNN models perform well when trained using a large dataset [43,44]. However, the majority of biomedical imaging datasets lack sufficient data. Therefore, data augmentation has been primarily used to address the above concerns which enhances variability in images and acts as a regularizer [45,46]. In the current study, data augmentation is applied over the training dataset using four transformations: horizontal flipping, rotation by an angle of 15 degrees (clockwise), scaling with 10%, and Gaussian noise with zero mean and variance of 0.25.

**Image Normalization:** To preserve numerical stability, normalization has been widely adopted in the CNN models. It also facilitates faster learning and stability in the gradient descent technique [47]. Therefore, we employed normalization that normalizes each image pixel by multiplying it by 1/255 so that it falls in a range, i.e., 0 to 1.

#### 2.2.2. Proposed Model

The DL models have revolutionized the field of AI because of their self-learning capabilities and superior classification performance in both binary and multi-class classification problems. Nevertheless, studies have been conducted recently in the field of biomedical image analysis either by implementing CNN models from scratch or by using transfer learning to achieve the best performance [48]. Both these methods have been recently studied for the classification of different lung infection diseases. However, most of these models deal with high computational costs and memory. Hence, the design of a lightweight CNN model is highly essential for the diagnosis of COVID-19 infection from CXR images. The proposed model includes three blocks: CBR blocks which contain convolution (CONV), batch normalization (BN), and rectified linear unit (ReLU) layer, each followed by a pooling layer and two fully connected (FC) layers at the end. A brief description of each layer is given below.

##### CONV Layer

This layer generates various feature maps by convolving the input volume with a set of filters [45]. It requires hyperparameters like the number of filters, size of the filter, stride, and padding.

##### BN Layer

BN layer helps to speed up the training process and normalizes the activation of the previous layer over a mini-batch [49]. It also acts as a regularizer to prevent overfitting issues. This layer is usually placed between CONV and ReLU layers.

##### ReLU Layer

This layer is essentially used to establish non-linearity in the model [45]. Due to its computational efficiency, ReLU has been extensively in DL models. It converts the negative values of inputs into zero and keeps positive values unchanged. The ReLU function can be defined mathematically as follows:

(1) ϕ(v)=max(0,v)

##### Pooling Layer

This layer down-samples the spatial size of the feature map and is generally placed in between consecutive CONV layers. It lessens the learnable parameters and computational cost, thereby handling the overfitting issues. Three popular pooling types are (i) max pooling, (ii) average pooling, and (iii) sum pooling; out of them, max pooling has been used in most of the recent studies [50]. Therefore, we used max pooling in this study.

##### FC Layer

The features extracted from the CONV layers are first flattened and then forwarded to the FC layers. They are placed at the end and are considered to be the final layers. These layers have similar characteristics to that of traditional neural networks. To handle the overfitting issue, a dropout layer is utilized between the FC layers. The last FC layer follows a softmax layer that yields a probability score with respect to every class which is used for classification.

#### 2.2.3. Proposed LW-CORONet Model

**Figure 4:**
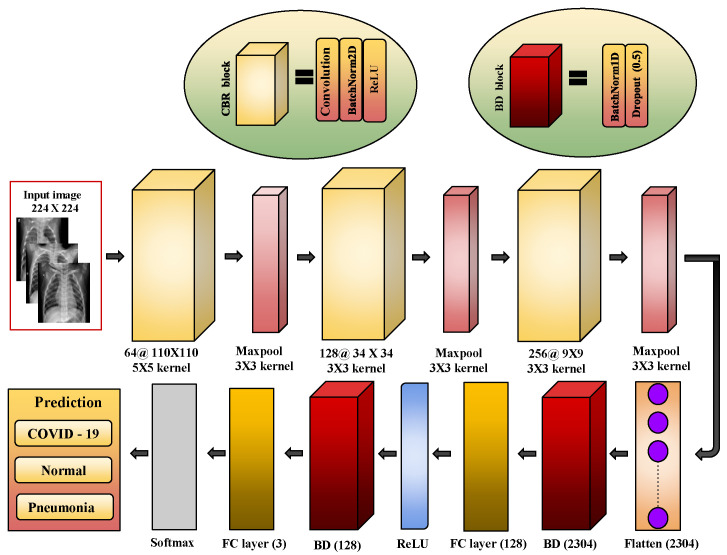
Illustration of proposed LW-CORONet model.

The proposed LW-CORONet model is depicted in Figure 4. It has three CBR blocks, each block includes a sequence of CONV, BN, and ReLU layers and follows a maxpool layer and two consecutive FC layers at the end. The 50% dropout is applied on each of the FC layers to avoid overfitting. Eventually, a softmax layer is placed. The prime objectives of designing the lightweight custom CNN model include the reduction of computational cost and the number of learning parameters, which helps in increasing its learning speed as compared to the state-of-art CNN methods.

The first CONV layer inputs a CXR image of size 224×224 and convolves it employing 64 filters of size 5×5 with stride 2 which produces an output volume of size 110×110×64. A max pooling operation with filter size 3×3 and a stride 3 is harnessed following the first CBR block, resulting in an output volume of size 36×36×64. Then, this output is fed to the second CBR block where 128 kernels of size 3×3 are used that yield an output volume of size 34×34×128. Further, a max pooling operation is employed to obtain an output of size 11×11×128. Similarly, in the third block, 256 filters of size 3×3 are applied followed by a max pooling operation to generate a volume of size 3×3×256. Then, this output volume is flattened and fed to a dense layer of 128 neurons. This layer is preceded by BN and dropout layers and followed by ReLU and BN layers. Finally, a soft-max layer is used at the end that produces the classification results. The learnable parameters involved in each layer and the detailed architecture is given in Table 2.

#### 2.2.4. Pre-Trained CNN Models with Transfer Learning

A brief overview of four pre-trained CNN architectures considered in this work with the concept of transfer learning (TL) is presented in this section. With TL, the pre-trained models that were trained on large-scale datasets like ImageNet are further trained to learn a similar task. Therefore, the learning process is computationally faster than learning a model from the scratch. It also performs well in the absence of huge training data. A large number of efforts have been made using TL for COVID-19 diagnosis due to the unavailability of a large number of samples. Notably, we fine-tune only the final layer while keeping the pre-trained weights the same. In this study, we evaluated four architectures: VGG-19 [33], ResNet-101 [34], DenseNet-121 [35], and Xception [36] and compared the impact of these models with our proposed model. Table 3 shows the architectural overview of each pre-trained model along with the proposed scheme.

## 3. Experimental Setup and Results

In this section, we present the experimental setup and results to verify the effectiveness of the proposed model. A set of experiments were performed using two datasets: Dataset-1 of 2250 frontal-view CXR images (pneumonia: 750, COVID-19: 750, and normal: 750) and Dataset-2 of 15,999 CXR images (pneumonia: 5575, COVID-19: 2358, and Normal: 8066). These images were first rescaled into a size 224×224. All CNN models were developed using the PyTorch toolbox and all experiments were conducted on the Google Colab GPU platform with NVIDIA Tesla T4 GPU of 16 GB RAM. The performance of both the suggested approach and pre-trained architectures was evaluated using 10-fold cross-validation (CV) for Dataset-1, wherein in each trial, one fold was utilized for testing, and the rest folds for training. From the training set, 20% of samples were randomly chosen for validation. For Dataset-2, results were evaluated based on the train-test division strategy reported by Wang et al. [22], wherein the test set contained 200 CXR images from each class and the remaining samples were retained for training the model, of which 20% are used for validation.

A different set of performance metrics such as sensitivity, specificity, precision, accuracy, and F1-score were used to assess each model. Furthermore, the heap-map results were computed using gradient-weighted class activation mapping (Grad-CAM) [51] to visually interpret the effectiveness of the model by highlighting the relevant regions. We evaluated the performance of the model in two different scenarios: the first scenario deals with three-class classification (pneumonia, COVID-19, and normal) and the second scenario deals with binary classification (Normal and COVID-19). The hyperparameter setting used in our study is presented in Table 4 which has been set empirically. Furthermore, a comparison analysis with pre-trained CNN architectures, namely VGG-19 [33], ResNet-101 [34], DenseNet-121 [35], and Xception [36] was done.

### 3.1. Results of the Proposed Model

The classification results obtained by the proposed model on both datasets are presented in this section.

#### 3.1.1. Results on Dataset-1

**Figure 5:**
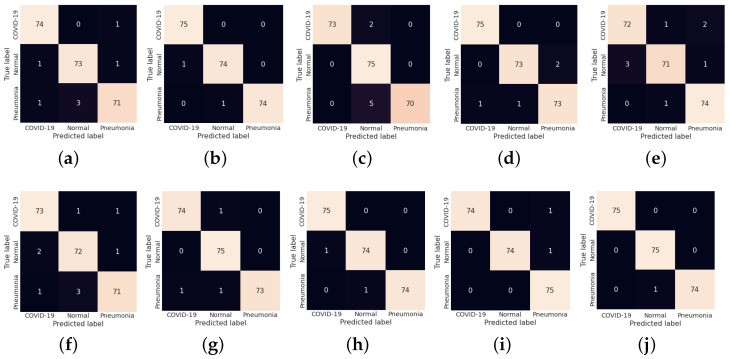
(**a**–**j**) Confusion matrix obtained by LW-CORONet fold-wise (fold 1 to fold 10) for multi-class classification case.

**Figure 6:**
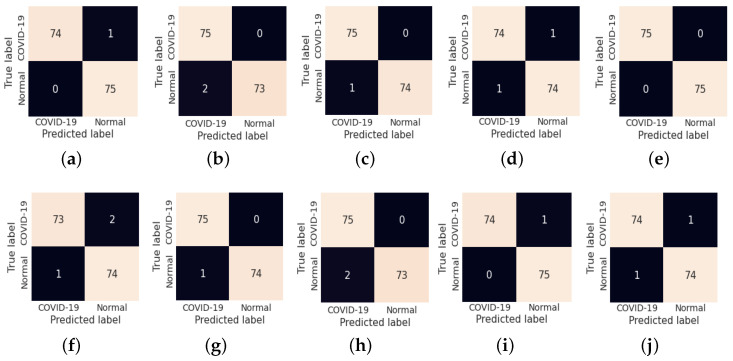
(**a**–**j**) Confusion matrix obtained by LW-CORONet fold-wise (fold 1 to fold 10) for binary classification case.

**Figure 7:**
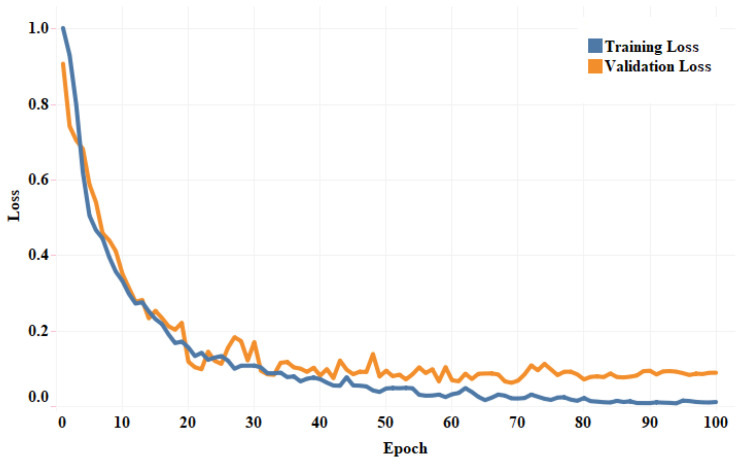
Illustration of the loss convergence plot for the first run of 10-fold CV.

**Figure 8:**
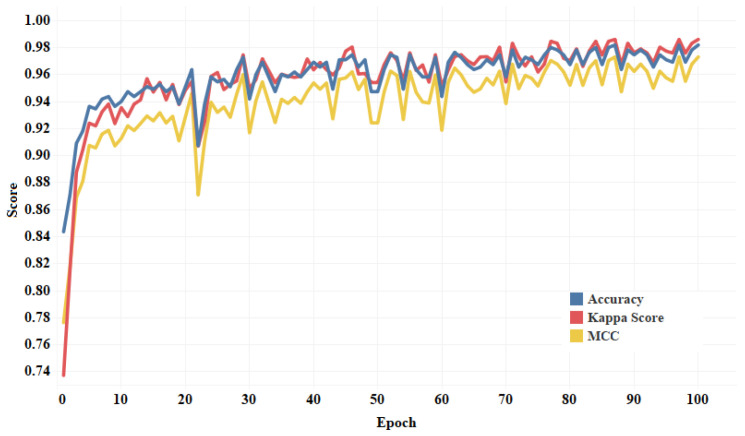
Graphical plot of Mathews correlation, kappa score, and accuracy for LW-CORONet model.

**Figure 9:**
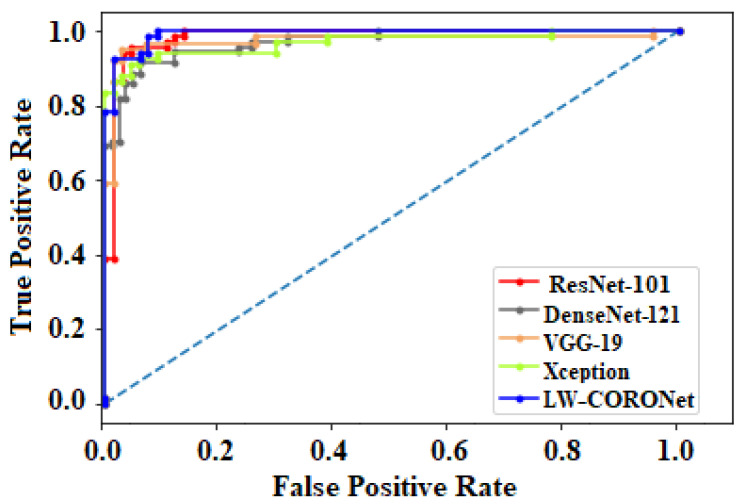
ROC curves of LW-CORONet and the pre-trained CNNs.

The fold-wise results in terms of accuracy, precision, sensitivity, and F1-score for Dataset-1 are shown in Table 5. The proposed model was also tested over binary classification tasks and the results are shown in Table 6. Notably, the results provided in each fold were average results computed over all three classes. The average accuracy of 98.67% and 99.00% was obtained for multi-class and binary-class classification tasks, respectively. The confusion matrices in each run of 10-fold CV for multi-class and binary class scenarios are exhibited in Figure 5 and Figure 6, respectively. Figure 7 shows the training and validation loss curves. It can be observed that the proposed model converges within 100 epochs. The plot is shown for a single run of a 10-fold CV. The Mathew correlation coefficient (MCC) and kappa score were computed as 0.9730 and 0.9860, respectively which indicate better prediction results for each class. The pictorial presentation of MCC and kappa score along with multi-class classification accuracy is depicted in Figure 8. These values were recorded at different epochs and computed for a single fold. Figure 9 shows the receiver operating characteristic (ROC) curves obtained by the suggested and pre-trained models for binary classification scenarios.

**Figure 10:**
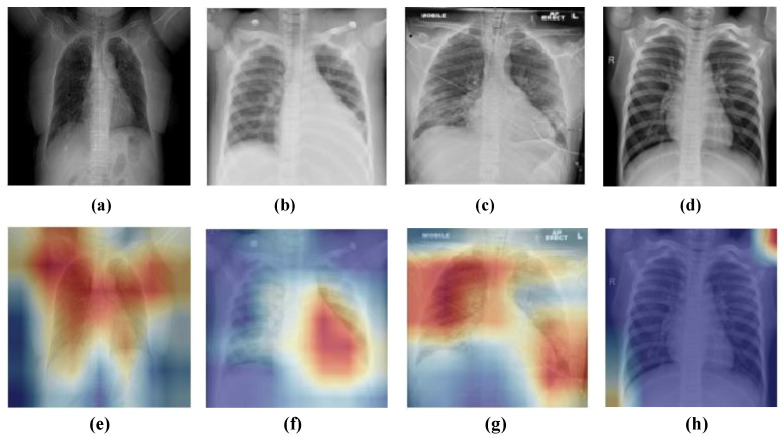
Grad-CAM visualization results of the proposed model on a few sample CXR images. (**a**–**d**) The top row indicates the original images. (**e**–**h**) The bottom row indicates the heat maps obtained by LW-CORONet.

Finally, heat maps were provided using Grad-CAM to verify the visual interpretability of the proposed model. Figure 10 illustrates the heat map results of a few sample normal and COVID-19 CXR images. The proposed model could locate the suspicious regions that indicated better interpretability of the classification results. Hence, it can be helpful to assist radiologists in their diagnosis. Figure 10h depicts the heat map of a normal sample where suspicious regions are not indicated.

The computational cost of the proposed model was evaluated in terms of time (in seconds). Our model takes around $8.5\times 10^{3}$ s in the training stage to converge, which is comparatively faster than training the state-of-the-art CNN architectures. While testing an image takes approximately 0.035 s using the proposed model.

#### 3.1.2. Results on Dataset-2

**Figure 11:**
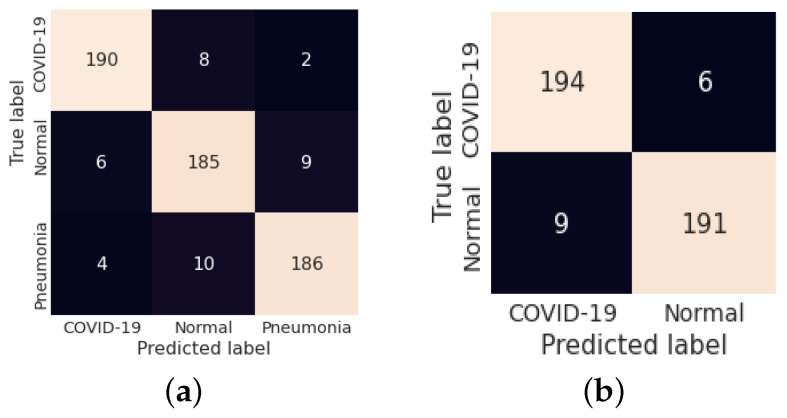
(**a**,**b**) Confusion matrix obtained for both cases on Dataset-2.

The performance of the proposed model is evaluated on Dataset 2. Table 7 shows the classification results for both the classification scenarios (multi-class and binary classification) for Dataset-2. The average accuracy of 95.67% and 96.25% was achieved for multi-class and binary classification, respectively. Figure 11 shows the confusion matrices for both scenarios.

### 3.2. Experiment on Different Hyperparameters

In this section, the impact of different hyperparameters, namely the number of epochs, batch size, learning rate, different optimizers, etc., is evaluated to identify the best classification performance. It is worth noting here that all experiments were conducted using Dataset-1.

#### 3.2.1. Impact of Optimization Techniques

In this experiment, we explore several optimization techniques such as SGD [52], Adam [53], RMSProp [54], and AdaGrad [55] to ascertain the best classification performance. Table 8 shows the detailed classification results using different optimizers. It can be seen that observed that the classification performance of the LW-CORONet with Adam optimizer is promising as compared to others. Therefore, all remaining experiments in this study were carried out using the Adam optimizer.

#### 3.2.2. Impact of Learning Rate

**Figure 12:**
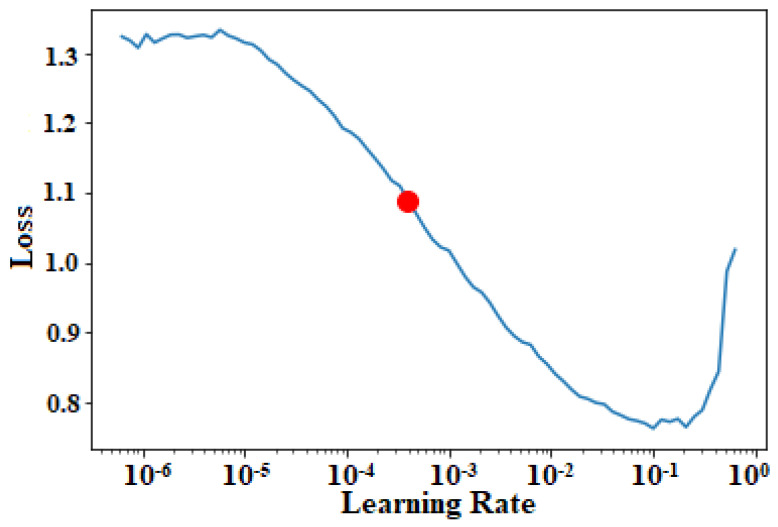
Plot between the learning rate and validation loss.

In this experiment, we investigated the impact of different learning rates, and the learning rate with minimum validation loss was chosen. Figure 12 illustrates a plot of learning rates versus validation loss. The point (represented in red dot) in the Figure 12 specifies the optimal learning rate from which the loss drops significantly, thereby, facilitating better classification performance.

#### 3.2.3. Impact of Different Batch Sizes

We evaluated the effect of different batch sizes in this experiment. The performance of LW-CORONet when trained with batch sizes of 32, 16, and 8 is tabulated in Table 9. These results showed that the proposed model with a batch size of 32 results in stable and higher testing performance.

### 3.3. Misclassification Results Analysis

**Figure 13:**
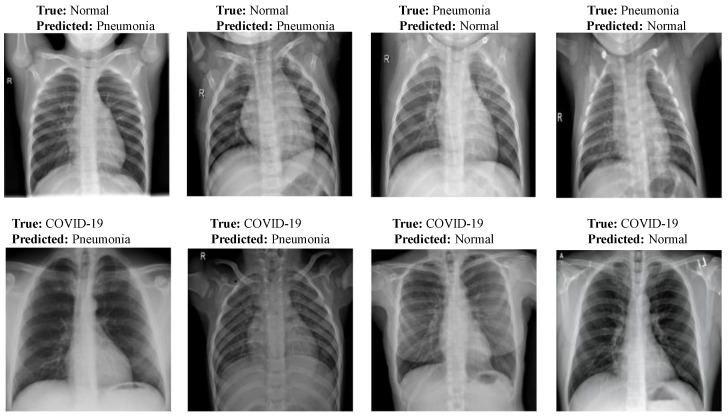
Sample misclassification outcomes of LW-CORONet.

Figure 13 shows few misclassification results yielded by LW-CORONet on Dataset-1. These errors possibly occurred because of the similar visual features among the CXR images of the three classes.

### 3.4. Comparative Analysis of Pre-Trained CNN Architectures and the Proposed Model

We performed a comparative analysis among the pre-trained classification architectures, VGG-19 [33], ResNet-101 [34], DenseNet [35], and Xception [36] and the proposed model. The detailed classification performance on Dataset-1 is tabulated in Table 10. It is worth mentioning here that these models were validated on the same images as done for the proposed model and the experimental setup was also the same. The proposed model required fewer parameters and memory but obtained a comparable or better performance compared to effective architectures like ResNet-101. All these pre-trained models were originally designed over ImageNet [56] dataset but, were fined tuned later using the CXR images to perform multi-class classification. We fine-tuned only the last layer of these models and initialized other layers with the pre-trained weights.

### 3.5. Comparison with Existing Approaches

The proposed model was compared against the existing DL-based schemes for automated COVID-19 detection in CXR images. Table 11 summarizes the obtained performance. It can be observed that the suggested model obtained promising results as compared with other approaches on Dataset-1 and achieved comparable or better performance even on the larger dataset (Dataset-2). The proposed model was validated using a relatively larger number of CXR samples compared to several recent studies [17,18,19,23,28,30,31,32]). Although studies in [20,21] employed datasets of comparable sizes, the COVID-19 class samples are only 76 and 180, respectively which imposes the class imbalance problem. Further, several studies were focused on solving either binary or multi-class classification tasks. But, the current study investigated both tasks and achieved an accuracy of 99.00% and 98.67% for binary and multi-class classification respectively on Dataset-1. Similarly, for Dataset-2, an accuracy of 96.25% and 95.67% was achieved for binary and multi-class classification, respectively.

### 3.6. Discussion

An automated DL-based model, LW-CORONet, is proposed for the effective detection of COVID-19 infection using CXR images. Of late, in the setting of COVID-19 detection, many DL-based studies have been performed; however, most of these studies were limited to smaller datasets and require huge memory space and higher computational costs. Hence, LW-CORONet was designed aiming to handle these issues. LW-CORONet was capable of learning discriminant features directly from CXR images while demanding fewer learning parameters and memory.

To verify the effectiveness of the proposed scheme, several extensive experiments were carried out using two larger datasets of 2250 and 15999 CXR samples from three categories such as normal, pneumonia, and COVID-19. This study also explored the impact of various hyperparameters: batch size, learning rate, and optimizers. A comparative analysis was done with four contemporary pre-trained classification networks and state-of-art approaches. The proposed scheme yielded a higher classification accuracy of 98.67% and 99.00% for multi-class and binary cases respectively on Dataset-1, whereas it is 95.67% and 96.25% on a larger dataset (Dataset-2). The proposed model is thus effective, lightweight, and hence can be utilized by radiologists for the early diagnosis of COVID-19 infection and pneumonia. The main advantages of the proposed model are as follows:

- The proposed LW-CORONet has only five learnable layers which promote the learning of high-level features automatically from the CXR samples.
- The proposed model is well-suited for both binary and multi-class classification scenarios and does not involve a hand-held feature extraction process.
- The proposed architecture demands very few parameters compared to other CNN models and thus, necessitates low computational cost.
- The proposed architecture is lightweight and uses less memory space.

The major drawback of this investigation is that the proposed model was trained with limited COVID-19 data due to the unavailability of a large-scale COVID-19 dataset.

## 4. Conclusions

This paper proposed, LW-CORONet, a novel model, for the early and accurate detection of COVID-19 infection from CXR images. The LW-CORONet was composed of five learnable layers with low computational power and memory space to extract detailed features. For the validation of the proposed scheme, an extensive set of experiments was carried out using two publicly available CXR datasets with larger COVID-19 samples. The effect of notable hyperparameters was verified on the proposed model to detect the best detection performance. Comparisons with pre-trained CNN models as well as current existing approaches revealed the superiority of the proposed approach in both multi-class and binary classification scenarios. Furthermore, the LW-CORONet model is better in terms of memory and computational cost. Overall, the suggested model is effective, lightweight, and hence can be suitable for clinicians for real-time COVID-19 diagnosis. However, in the future, the performance of LW-CORONet is suggested to be verified over a big and diverse dataset with more COVID-19 samples. Also, the lungs affected regions for both pneumonia and COVID-19 cases can be identified simultaneously with the classification of CXR images.

## Figures and Tables

**Table 1:** Description of the datasets.

Dataset	Number of CXR Images			Total
	Normal	Pneumonia	COVID-19	
Dataset-1	750	750	750	2250
Dataset-2	8066	5575	2358	15,999

**Table 2:** Detail configuration of the proposed LW-CORONet.

Layer	Activation Size	Parameters	Stride	Kernel Size	Filter
Input	(3, 224, 224)	0	-	-	-
CONV	(64, 110, 110)	4864	2	5×5	64
BN	(64, 110, 110)	128	-	-	-
ReLU	(64, 110, 110)	0	-	-	-
MaxPool	(64, 36, 36)	0	3	3×3	-
CONV	(128, 34, 34)	73,856	1	3×3	128
BN	(128, 34, 34)	256	-	-	-
ReLU	(128, 34, 34)	0	-	-	-
MaxPool	(128, 11, 11)	0	3	3×3	-
CONV	(256, 9, 9)	295,168	1	3×3	256
BN	(256, 9, 9)	512	-	-	-
ReLU	(256, 9, 9)	0	-	-	-
MaxPool	(256, 3, 3)	0	3	3×3	-
BN	2304	4068	-	-	-
Dropout (0.5)	2304	0	-	-	-
FC	128	295,040	-	-	-
ReLU	128	0	-	-	-
BN	128	256	-	-	-
Dropout (0.5)	128	0	-	-	-
Softmax	3	387	-	-	-
		Total ≊ 0.68 million			

**Table 3:** Architectural details of pre-trained CNN architecture.

Architecture	Layers	Input Layer Size	Output Layer Size	Parameters
ResNet-101	101	(224,224,3)	(3,1)	$44.1\times 10^{6}$
VGG-19	19	(224,224,3)	(3,1)	$143\times 10^{6}$
DenseNet-121	121	(224,224,3)	(3,1)	$7.5\times 10^{6}$
Xception	71	(299,299,3)	(3,1)	$22.3\times 10^{6}$
Proposed LW-CORONet	5	(224,224,3)	(3,1)	$0.68\times 10^{6}$

**Table 4:** Illustration of hyperparameter setting used in this study.

Hyperparameter	Value
Batch size	32
Learning rate	0.0005
Epochs	100
Optimizer	Adam
Loss function	Categorical cross-entropy

**Table 5:** Fold-wise multi-class classification performance (in %) on Dataset-1.

Fold	Accuracy	Precision	Sensitivity	Specificity	F1-Score	COVID-19 Class Accuracy
1	97.92	96.89	96.89	98.44	96.88	98.66
2	99.41	99.12	99.11	99.56	99.11	99.56
3	97.97	97.15	97.03	98.49	96.99	99.11
4	98.82	98.65	97.33	99.33	97.88	99.56
5	97.63	96.45	96.44	98.22	96.44	97.33
6	97.35	96.02	96.02	98.01	96.01	97.78
7	99.11	98.69	98.67	99.33	98.67	99.11
8	99.41	99.12	99.11	99.56	99.11	99.56
9	99.41	99.13	99.11	99.56	99.11	99.11
10	99.70	99.56	99.56	99.78	99.56	100.00
Average	**98.67**	**98.08**	**97.93**	**99.03**	**97.98**	**98.98**

**Table 6:** Fold-wise binary classification performance (in %) on Dataset-1.

Fold	Accuracy	Precision	Sensitivity	Specificity	F1-Score	COVID-19 Class Accuracy
1	99.33	100.00	98.67	100.00	99.33	98.67
2	98.67	97.40	100.00	97.33	98.68	100.00
3	99.33	98.68	100.00	98.67	99.33	100.00
4	98.67	98.67	98.67	98.67	98.67	98.67
5	100.00	100.00	100.00	100.00	100.00	100.00
6	98.00	98.65	97.33	98.67	97.99	97.33
7	99.33	98.68	100.00	98.67	99.34	100.00
8	98.67	97.40	100.00	97.33	98.68	100.00
9	99.33	100.00	98.67	100.00	99.33	98.67
10	98.67	98.67	98.67	98.67	98.67	98.67
Average	**99.00**	**98.82**	**99.20**	**98.80**	**99.00**	**99.20**

**Table 7:** Classification performance (in %) of LW-CORONet on Dataset-2.

Task	Accuracy	Precision	Sensitivity	Specificity	F1-Score	COVID-19 Class Accuracy
Multi-class	95.67	93.51	93.50	96.75	93.50	95.00
Binary	96.25	95.57	97.00	95.50	96.28	97.00

**Table 8:** Classification results (in %) obtained by various optimizers.

Optimizer	Accuracy	Precision	Sensitivity	Specificity	F1-Score
SGD	98.18	97.55	97.42	98.61	97.39
RMSProp	97.94	97.20	97.05	98.43	97.03
AdaGrad	98.55	98.01	97.91	98.89	97.90
**Adam**	**98.67**	**98.08**	**97.93**	**99.03**	**97.98**

**Table 9:** Accuracy of LW-CORONet with different batch sizes.

Model	Batch Size		
	32	16	8
Proposed LW-CORONet	98.67	98.03	97.26

**Table 10:** Classification performance comparison (in %) among pre-trained CNNs.

Model	Accuracy	Precision	Sensitivity	Specificity	F1-Score	Parameter	Memory
ResNet-101	98.19	97.29	97.29	98.64	97.29	44.1 M	168 MB
VGG-19	97.81	96.76	96.71	98.36	96.71	143 M	547 MB
DenseNet-121	97.45	96.16	96.29	98.10	96.20	7.5 M	31 MB
Xception	96.97	95.43	95.53	97.74	95.46	22.3 M	84 MB
**LW-CORONet**	**98.67**	**98.08**	**97.93**	**99.03**	**97.98**	**0.68 M**	**6 MB**

**Table 11:** Comparison of proposed model with existing COVID-19 detection approaches.

Reference	Method	Number of CXR Samples	Accuracy (%)	
			Binary Class	Multi-Class
Hemdan et al. [18]	COVIDX-Net	50	90.00	–
		($C_{1}$: 25 and $C_{2}$: 25)		
Narin et al. [19]	ResNet-50	100	98.00	–
		($C_{1}$: 50 and $C_{2}$: 50)		
Ozturk et al. [17]	DarkCovidNet	1125	98.08	87.02
		($C_{1}$: 125, $C_{2}$: 500 and $C_{3}$: 500)		
Ucar and Korkmaz [20]	Bayes-SqueezeNet	5949	–	98.30
		($C_{1}$: 76, $C_{2}$: 1583 and $C_{3}$: 4290)		
Rahimzadeh and Attar [21]	Xception and ResNet50V2	15085	–	91.40
		$C_{1}$: 180, $C_{2}$: 6054 and $C_{3}$: 8851		
Toğaçar et al. [23]	SqueezeNet and MobileNetV2	458	–	98.25
	SVM	($C_{1}$: 295, $C_{2}$: 65 and $C_{3}$: 98)		
Nayak et al. [32]	ResNet-34	406	98.33	–
		($C_{1}$:203 and $C_{2}$: 203)		
Toramana et al. [24]	CapsNet	3150	97.24	84.22
		$C_{1}$: 1050, $C_{2}$: 1050 and $C_{3}$: 1050		
Chaudhary and Pachori [28]	FBSED and Inception-ResNet-v2	1446	-	93.06
		$C_{1}$: 482, $C_{2}$: 482 and $C_{3}$: 482		
Bhattacharyya et al. [30]	VGG-19 and BRISK	1030	-	96.60
		$C_{1}$: 342, $C_{2}$: 341 and $C_{3}$: 347		
Jyoti et al. [31]	ResNet-50 and MCA-TQWT	5275	94.64	-
		$C_{1}$: 2409 and $C_{2}$: 2866		
**Proposed model**	**LW-CORONet**	**Dataset-1: 2250**	**99.00**	**98.67**
		($C_{1}$: 750, $C_{2}$: 750 and $C_{3}$: 750)		
		**Dataset-2: 15,999**	**96.25**	**95.67**
		($C_{1}$: 2358, $C_{2}$: 8066 and $C_{3}$: 5575)		

*C*_1_: COVID-19, *C*_2_: Normal, *C*_3_: Pneumonia.

## Data Availability

In this study, we used two publicly available COVID-19 chest X-ray datasets: Dataset-1 (https://figshare.com/articles/COVID-19_Chest_X-RayImage_Repository/12580328) and Dataset-2 (https://github.com/lindawangg/COVID-Net).
